# Effect of storage on the chemical composition, microbiological load, and sensory properties of cassava starch-based custard powder

**DOI:** 10.1002/fsn3.235

**Published:** 2015-04-09

**Authors:** Wasiu Awoyale, Lateef O Sanni, Taofik A Shittu, Mojisola O Adegunwa

**Affiliations:** 1 Department of Food, Agricultural and Bioengineering, Kwara State UniversityMalete, Nigeria; 2 Department of Food Science and Technology, Federal University of AgricultureAbeokuta, Nigeria; 3 Department of Hospitality and Tourism, Federal University of AgricultureAbeokuta, Nigeria

**Keywords:** Chemical composition, custard, microbiological load, package, sensory quality, storagestorage

## Abstract

The effect of storage on the chemical, microbiological, and sensory properties of cassava starch-based custard powder (CbCP) blends as mixture of yellow-fleshed cassava root starch (YfCRS) (90–98%) and whole egg powder (WEP) (2–10%) was investigated. These were prepared using central composite rotatable design, and separately packaged in polyvinyl chloride plastic can and stored in storage box (30 ± 2°C). The chemical and microbiological analyses of the stored CbCP were evaluated at 3 weeks intervals, while the sensory property was determined at 6 weeks interval for 24 weeks. The result showed that the protein, fat, and the total-*β*-carotene contents of the CbCP decreased significantly (*P* ≤ 0.001) after storage while moisture content and microbiological load increased. All the CbCP sensory attributes were accepted at the end of storage, except taste and color. The CbCP gruel prepared from 94% YfCRS: 0.34% WEP and 90% YfCRS: 2% WEP blends were the most acceptable after storage.

## Introduction

Custard powder is a fine particulate food product made from corn starch with the addition of salt, flavor, and color, and with or without the inclusion of egg solids, vitamins, and minerals. This custard powder could serve as supplement for infant's feeding, consumed as breakfast meal by many and could be regarded as food of choice for the sick (Okoye et al. 2008). However, since Nigeria is the largest producer of cassava root, the use of starch extracted from yellow-fleshed cassava root, might reduce over dependence on imported corn starch for custard powder production. Hence, the use of yellow-fleshed cassava root starch (YfCRS) in this study. The production of custard powder from this YfCRS might be very poor in nutrients especially protein. Consistent consumption of such food without adequate protein intake might eventually lead to malnutrition. Therefore, supplementation of this food with high-quality animal protein product, such as whole egg powder (WEP) might improve its protein quality and quantity. It is also envisaged that the interaction of these components (YfCRS and WEP) might institute some changes in the product during storage. During storage, one or more food characteristics can reach an undesirable state and consequently the consumer may reject the product or the product can be detrimental to the health of the consumer. Teniola (2003) stated that packaging materials used for commercial sale of custard powder in Nigeria include polyvinyl chloride (PVC) containers and laminated paperboard cartons. This is because these materials are easily available, cheap, has high tensile strength and with low permeability to moisture, gases, and odor (Kadam et al. 2008). Awoyale et al. (2013) also reported that *ogi* powder packed in PVC plastic could be stored at a temperature range of between 27 to 30°C and relative humidity range of 58 to 66%, for good product quality. However, the effect of rigid PVC package on the chemical composition, microbiological load, and sensory properties of stored CbCP has not been documented. Additionally, there is presently no information on the chemical composition and sensory properties of CbCP made from the blends of YfCRS and WEP, and how these properties changes with storage.

Therefore, this work aimed at evaluating the effect of storage at room temperature on the chemical composition, microbiological load, and sensory properties of CbCP, using an airtight PVC container has packaging material.

## Materials and Methods

### Production of yellow-fleshed cassava root starch

**Figure 1:**
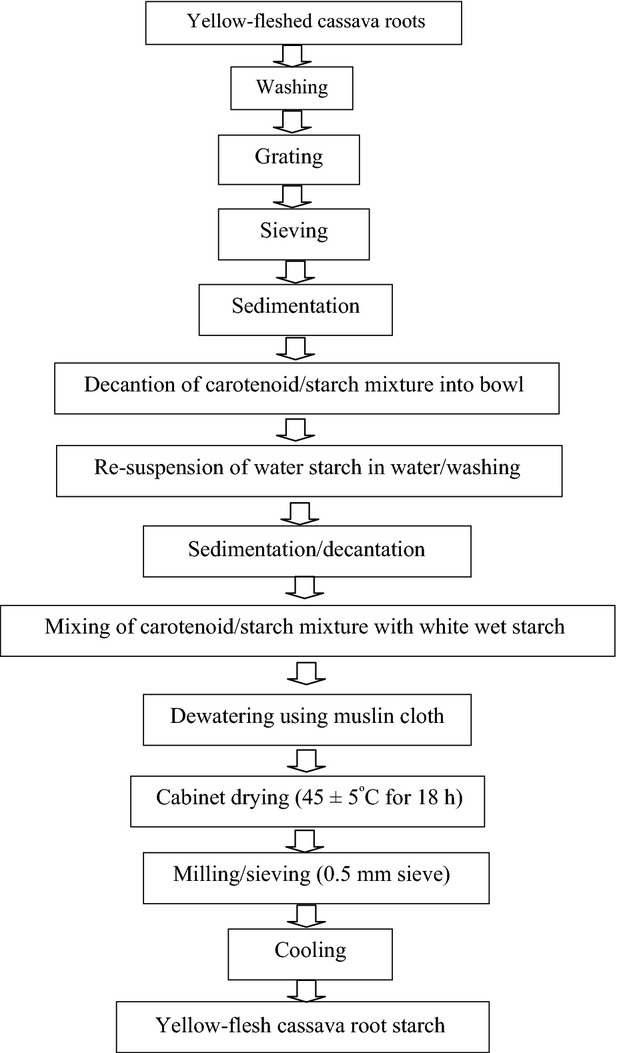
Flowchart of Yellow-fleshed Cassava Root Starch production (Modified from Oyewole and Obieze 1995).

The yellow-fleshed cassava root starch (YfCRS) was produced using the traditional method of starch extraction as described by Oyewole and Obieze (1995) (Fig.1) with modification. Freshly harvested YfCR obtained from the International Institute of Tropical Agriculture (IITA) Ibadan, was peeled, washed in water, and grated with an electric motor powered mechanical grater (locally fabricated by NOBEX Lagos, Nigeria limited). The resultant pulp was immediately sieved through a muslin cloth and suspended in water. This separates the fibrous and other coarse root material from the starch pulp. The starch pulp was allowed to settle for 4–6 h before decanting. The supernatant containing a mixture of yellow pigment and starch was decanted into a bowl and the thick sediment was the wet starch. The wet starch was reconstituted in water for washing twice, allowed to settle, after which the water was decanted and the starch mixed with the yellow pigment/starch mixture, pressed, and dried using a convectional cabinet dryer at 45 ± 5°C for 18 h. It was then allowed to cool to room temperature (30 ± 2°C), milled, and packaged in polythene nylon bag prior to further studies.

### Production of whole egg powder

**Figure 2:**
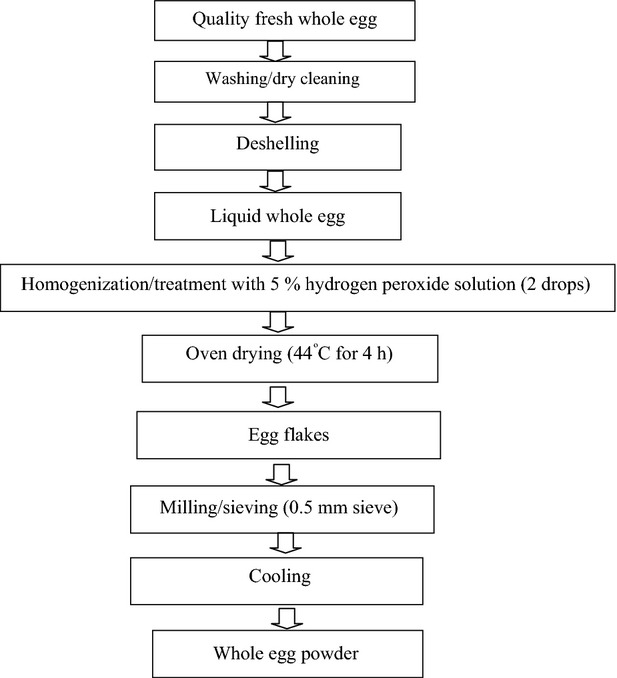
Flowchart of Whole Egg Powder production (Ndife et al. 2010).

The whole egg powder (WEP) was produced as described by Ndife et al. (2010) (Fig.2). Quality whole eggs were carefully washed, dry cleaned, deshelled, and properly homogenized with a metal whisk during which two drops of hydrogen peroxide solution was added to free the products from viable *salmonella* microorganism and to prevent browning. The sample was oven dried at 44°C for 4 h and allowed to cool to room temperature (30 ± 2°C). The egg flakes was scooped, milled, and sieved with a 60-mm mesh and then packaged in polythene nylon bag for further use.

### Experimental design for the preparation of cassava starch-based custard powder

**Figure 3:**
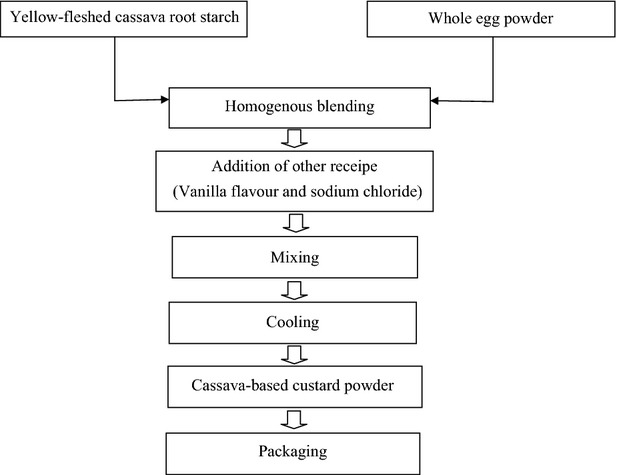
Flowchart of Cassava-based Custard Powder production.

Central composite rotatable design of Design-expert (Version 7.0) (Design-Expert 2005) was used to determine the experimental design and the ingredients combination levels for the CbCP formulation. The two basic ingredients incorporated in the custard were YfCRS (90–98%) and WEP (2–10%). This gave rise to 13 runs with five central points (Table1). The CbCP was prepared as shown in Figure3.

**Table 1:** Central composite design of the yellow-fleshed cassava root starch (YfCRS) and whole egg powder (WEP) combinations for the preparation of cassava starch-based custard powder

Trial	Coded values		Actual values	
	YfCRS (x_1_)	WEP (x_2_)	YfCRS	WEP
1	0	0	94.00	6.00
2	0	0	94.00	6.00
3	−1	+1	90.00	10.00
4	+1	+1	98.00	10.00
5	0	−*α*	94.00	0.34
6	0	0	94.00	6.00
7	+1	−1	98.00	2.00
8	+*α*	0	99.66	6.00
9	−*α*	0	88.34	6.00
10	−1	−1	90.00	2.00
11	0	0	94.00	6.00
12	0	0	94.00	6.00
13	0	+*α*	94.00	11.66

### Storage studies of cassava-based custard powder

The cassava starch-based custard powder (100 g) was properly weighed and packaged in PVC plastic can (19 × 13 cm diameter) and closed with the lid. This was stored at room temperature (30 ± 2°C) to determine the storage stability for 24 weeks in a storage box. The temperatures and relative humidities of the storage box were monitored with a thermo-hygrometer before sample collection (Table2). The chemical properties including the microbiological counts of the samples were determined every 3 weeks, while the sensory properties were done at 6 weeks interval for the 24 weeks of storage.

**Table 2:** Temperatures and Relative humidities of the storage box for the storage periods

Storage periods (Weeks)	Temperature (^o^C)	Relative Humidity (%)
0	29.50	63.90
3	29.60	64.00
6	29.70	69.00
9	29.40	75.00
12	30.10	81.00
15	29.30	84.00
18	29.90	75.00
21	29.50	80.00
24	30.20	97.00

### Moisture content

The moisture content was determined using AOAC (2000) method. About 3 g of sample was weighed into a preweighed clean dried dish, after which the dish was placed in a well-ventilated oven (draft air Fisher Scientific Isotemp® Oven model 655F, Springfield, USA) maintained at 103 ± 2°C for 24 h. The loss in weight was recorded as moisture content.

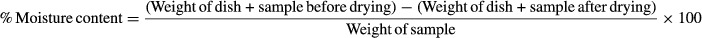


### Ash content

This was determined using the method of AOAC (2000). It involves burning off moisture and all organic constituents at 600°C for 5 h in a furnace (United State of America (USA)). The weight of the residue after incineration was then recorded as the ash content.

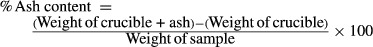


### Crude protein

The crude protein was determined by Kjeldahl method using Kjeltec™ model 2300 protein analyzer, as described in Foss Analytical AB (2003). About 0.2 g of sample was digested at 420°C for 1 h to liberate the organically bound nitrogen in the form of ammonium sulfate. The ammonia in the digest (ammonium sulfate) was then distilled off into a boric acid receiver solution, and then titrated with standard hydrochloric acid. A conversion factor of 6.25 was used to convert from total nitrogen to percentage crude protein (displayed on the screen of the protein analyzer)

### Fat content

This was determined using AOAC (2000) method. Crude fat was extracted from 3 g of the sample with hexane using a fat extractor (Soxtec System HT-2 fat extractor, Apeldoorn, The Netherlands), and the solvent was evaporated off to get the fat. The difference between the initial and final weight of the extraction cup was recorded as the crude fat content.

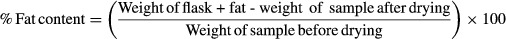


### pH value

Samples (5 g) was suspended in deionized water for 5 min at a ratio of 1:5 (w/w) and pH measured using a digital pH meter (Orion Research Inc., Model 720A, USA) as reported by Sanni, (1999).

### Total β-carotene content

The total *β*-carotene content of yellow-fleshed cassava root starches was determined as reported by Rodriguez-Amaya and Kimura (2004).

### Microbiological load

The cassava starch-based custard powder was subjected to microbiological analyses (total bacteria, mold, and yeast counts) immediately after production and during storage. Each CbCP sample (1 g) was serially diluted by dissolving in 9 mL of distilled water. Using the pour plate method, each diluent was plated out on a plate count agar for bacterial count, potato dextrose agar to which 0.01% chloramphenicol had been added to inhibit bacteria growth for yeast count and Sabouraud dextrose agar for mold counts. The plates were incubated at 37°C for 48 h for bacterial growth and at 27°C for 3 days for yeasts and mold growth (Amankwah et al. 2009).

### Preparation of cassava starch-based custard powder for sensory evaluation

The custard gruel was prepared by mixing 20 g of CbCP with 100 mL of tap water in a small plastic bowl. Thereafter, 80 mL of boiling water was added to each of the suspended sample and mixed to produce hot gruel. After preparation, a teaspoonful of sucrose (9 g) was added to each of the gruel to improve its taste. The samples of gruel produced were then served hot to the panelist (Okoye et al. 2008). A 9-point hedonic preference scale was used to test the month-feel, taste, color, flavor, appearance, and overall acceptability of the CbCP gruel using commercial vanilla-flavored (Gold™ brand, Lagos, Nigeria.) custard as control, where nine corresponds to like extremely and 1 to dislike extremely. Twelve trained panelists were selected from the staff and graduate students of International Institute of Tropical Agriculture (IITA), Ibadan, and screened with respect to their interest and ability to differentiate food sensory properties as described by Iwe (2002).

### Data analysis

All analyses were carried out in triplicates and obtained data were subjected to one-way analysis of variance (ANOVA) using Statistical Analysis System (SAS) package, version 9.1 (SAS Institute, Inc., Cary, NC) (SAS 2008). Means were separated using fisher's protected least significant difference test.

## Results and Discussions

### Effect of storage on the chemical composition of cassava starch-based custard powder

Product storage stability is controlled by three factors; product characteristics, the environment to which the packaged product is exposed during distribution and the properties of the package (Robertson 2000). This might be altered by changing the food composition and form, the environment to which it is exposed, or its packaging system (Harte and Gray 1987). Polar polymers are excellent barriers to nonpolar permeate molecules (such as oxygen) but poor barriers to polar permeate molecules (such as water vapor). Therefore, an increase in relative humidity will cause an increase in the permeability of polar polymers (Smith and Hui 2004). This might be responsible for the significant effect of blend ratio, storage conditions, and interactions between blend ratio and storage conditions on the chemical composition and microbiological load of the CbCP after storage (Table3).

**Table 3:** Effect of storage on the chemical composition of cassava-based custard powder

Parameters	Protein (%)	Ash (%)	MC (%)	Fat (%)	pH	Total -carotene
Storage period (Weeks)						
0	7.92 (14.77)^a^	1.68 (0.70)^b^	8.50 (1.58)^b^	8.38 (9.46)^a^	5.22 (1.21)^a^	0.1777 (0.05)^a^
3	7.92 (14.77)^a^	2.06 (0.78)^a^	8.17 (1.59)^c^	8.36 (9.46)^c^	5.01 (1.18)^c^	0.1750 (0.05)^b^
6	7.92 (14.77)^a^	1.66 (0.69)^bc^	7.80 (1.13)^d^	8.37 (9.52)^b^	5.06 (1.09)^b^	0.1723 (0.05)^c^
9	7.90 (14.76)^c^	1.61 (0.71)^c^	7.87 (1.15)^d^	8.37 (9.45)^ab^	4.92 (1.04)^d^	0.1718 (0.05)^cd^
12	7.91 (14.77)^bc^	2.06 (0.78)^a^	8.43 (1.22)^b^	8.38 (9.50)^ab^	5.01 (1.18)^c^	0.1696 (0.05)^cd^
15	7.90 (14.76)^c^	1.66 (0.69)^bc^	8.18 (1.61)^c^	8.34 (9.49)^d^	5.06 (1.09)^b^	0.1696 (0.05)^cd^
18	7.90 (14.75)^c^	1.61 (0.71)^c^	7.82 (1.14)^d^	8.34 (9.48)^d^	4.92 (1.04)^d^	0.1686 (0.05)^cd^
21	7.90 (14.75)^c^	2.06 (0.78)^a^	9.27 (1.68)^a^	8.33 (9.51)^e^	5.01 (1.18)^c^	0.1677 (0.05)^d^
24	7.91 (14.76)^bc^	1.66 (0.69)^bc^	9.32 (1.72)^a^	8.31 (9.51)^f^	5.06 (1.09)^b^	0.1614 (0.04)^e^
Blend ratios						
94% YfCRS: 0.34% WEP	0.74 (0.01)^j^	1.78 (0.86)^cd^	8.91 (0.83)^c^	3.70 (0.01)^j^	4.31 (0.29)^h^	0.1899 (0.01)^b^
90% YfCRS: 2% WEP	1.72 (0.02)^h^	1.72 (0.23)^de^	9.35 (0.58)^ab^	4.45 (0.10)^h^	4.26 (0.10)^i^	0.1763 (0.02)^de^
98% YfCRS: 2% WEP	1.63 (0.01)^i^	1.68 (0.24)^ef^	9.51 (0.89)^a^	4.30 (0.07)^i^	4.26 (0.11)^i^	0.1896 (0.01)^b^
88.34% YfCRS: 6% WEP	3.93 (0.02)^e^	1.77 (0.30)^cd^	8.83 (0.90)^c^	5.73 (0.06)^e^	4.61 (0.30)^g^	0.1913 (0.01)^b^
94% YfCRS: 6% WEP	3.73 (0.02)^f^	1.72 (0.27)^de^	9.22 (0.42)^b^	5.67 (0.07)^f^	4.86 (0.15)^e^	0.1794 (0.01)^c−e^
99.66% YfCRS: 6% WEP	3.55 (0.02)^g^	1.41 (0.23)^g^	8.40 (1.12)^d^	5.56 (0.04)^g^	4.80 (0.11)^f^	0.1839 (0.01)^c^
90% YfCRS:10% WEP	5.85 (0.02)^c^	1.60 (0.44)^f^	8.55 (0.79)^d^	7.02 (0.09)^c^	5.27 (0.16)^c^	0.1961 (0.01)^a^
98% YfCRS:10% WEP	5.43 (0.05)^d^	1.83 (0.35)^c^	8.31 (0.74)^d^	6.76 (0.03)^d^	5.24 (0.11)^d^	0.1898 (0.02)^b^
94% YfCRS: 11.66% WEP	6.41 (0.02)^b^	2.04 (0.39)^b^	8.51 (0.96)^d^	7.47 (0.10)^b^	5.28 (0.09)^b^	0.1755 (0.01)^e^
TMS01/1368 YfCRS	0.56 (0.01)^k^	0.59 (0.13)^h^	7.71 (1.11)^e^	3.59 (0.03)^k^	4.18 (0.18)^j^	0.1806 (0.00)^cd^
WEP	53.48 (0.04)^a^	3.48 (0.15)^a^	4.80 (1.32)^f^	37.63 (0.07)^a^	8.25 (0.26)^a^	0.0222 (0.01)^f^
Mean	7.91	1.78	8.37	8.35	5.03	0.1704
CV (%)	0.19	8.06	5.59	0.27	0.36	5.06
*P* blend ratios	***	***	***	***	***	***
*P* storage	***	***	***	***	***	***
*P* storage × blend ratios	***	***	***	***	***	***

*** *P* ≤ 0.001, ()-Standard deviation, MC-Moisture content.

Means with different superscript on the same column are significantly different at *P* ≤ 0.05.

The moisture content (MC) of the CbCP increased significantly (*P* ≤ 0.001) from 8.50% before storage to 9.32% at the end of storage (Table3). The 98% YfCRS and 2% WEP blends had the highest MC while 98% YfCRS and 10% WEP had the lowest. This increase in MC might be attributed to the increase at relative humidity in ambient storage conditions after 24 weeks of storage (Smith and Hui 2004). Nevertheless, the MC of all the CbCP at the end of storage was below the recommended safe level (12–13%) for storage of flour (FAO 2000).

The protein content significantly (*P* ≤ 0.001) reduced from 7.92% (94% YfCRS: 11.66% WEP) before storage to 7.91% (94% YfCRS: 0.34% WEP) after storage (Table3). This might be associated with the absorption of moisture from the storage atmosphere that further accelerated microbiological growth. The microbes might have used part of the CbCP protein as nutrient for growth during the exponential phase (Jay 2000; Adams and Moss 2005), hence, the significant (*P* ≤ 0.01) negative correlation (*r* = −0.61) between protein content and bacterial counts (Table4). However, CbCP produced from 94% YfCRS and 11.66% WEP retains its highest protein content at the end of storage (Table3).

**Table 4:** Pearson correlations of the initial chemical composition and the microbiological count of Cassava-based Custard Powder after 24 weeks of storage

Parameters	Protein	Ash	Moisture	pH	Fat	*β*-carotene	Bacterial	Yeast	Mold
Protein	1.00								
Ash	0.27	1.00							
Moisture	−0.15	−0.41*	1.00						
Ph	1.00**	0.32	−0.19	1.00					
Fat	1.00**	0.27	−0.15	1.00**	1.00				
*β*-carotene	−1.00**	−0.27	0.14	−1.00**	−1.00**	1.00			
Bacterial	−0.61**	0.23	0.21	−0.58**	−0.61**	0.61**	1.00		
Yeast	0.74**	−0.02	0.02	0.71**	0.74**	−0.74**	−0.73**	1.00	
Mold	0.36	0.36	−0.16	0.38	0.36	−0.36	−0.13	0.36	1.00

**P ≤ 0.01, *P ≤ 0.05.

There was a significant (*P* ≤ 0.001) decrease in the fat content of the CbCP from 8.38% before storage to 8.31% at the end of the storage period (Table3). This decrease in fat content might be associated with the relative humidity of the storage conditions, which could have stimulated the activity of lipase and thus, splits up fat into free fatty acids and glycerol resulting in reduction in total fat content of the product (Akhtar et al. 2005). Additionally, the microbes might have used up part of the fat as nutrient for growth during the exponential phase (Jay 2000; Adams and Moss 2005), thus, the reason for the significant (*P* ≤ 0.01) negative correlation (*r* = −0.61) between the fat content and bacterial counts of the stored CbCP (Table4). Nevertheless, 94% YfCRS and 11.66% WEP blends maintain its high fat content at the end of the 24 weeks of storage (Table3).

The pH value of the CbCP reduced significantly (*P* ≤ 0.001) from 5.22 before storage to 5.06 at the 24 weeks storage period. This might be an indication that the product becomes more acidic with storage. This rise in acidity may be assigned to the accumulation of linoleic acids and other amino acids during storage, which are oxidized later (Kent and Evers 1994). Hence, significant (*P* ≤ 0.01) positive correlations exist between pH value, and the fat and protein contents of the stored CbCP (Table4). However, the CbCP with the highest WEP content (94% YfCRS: 11.66% WEP) had the highest pH value at the end of storage (Table3).

The total *β*-carotene content of the CbCP significantly (*P* ≤ 0.001) reduced at the end of storage (Table3). Rodriguez-Amaya (1999) reported that dehydrated products are considered more likely to undergo carotenoid degradation during storage because of the increase in surface area and porosity vis-à-vis the storage conditions and the packaging material permeability to oxygen and moisture. This statement was in agreement with the present work, as the total *β*-carotene content of the CbCP decreased with storage. However, custard powder with blend ratio of 90% YfCRS and 10% WEP had the highest *β*-carotene contents at the end of storage (Table3). Additionally, the total *β*-carotene content of the CbCP before storage had a significant positive correlation with bacterial count (*P* ≤ 0.01, *r* = 0.61) and negative for yeast count (*P* ≤ 0.01, *r* = −0.74) (Table4).

### Effect of storage on the microbiological count of the cassava starch-based custard powder

The total bacterial, yeast, and mold counts of the CbCP increased as the storage period increased (Table5). The blend ratio of 94% YfCRS and 6% WEP had the highest bacterial count, 94% YfCRS and 11.66% WEP had the highest yeast count and the mold count was highest in 98% YfCRS and 10% WEP blends at the end of the 24 weeks of storage (Table5). The increased microbiological counts of the stored CbCP could be attributed to the moisture absorption during storage. The possible use of the stored CbCP protein and fat as nutrients during the exponential growth phase may have contributed to the increase in microbiological count, since there exists a significant negative correlation between bacterial counts and the protein and fat contents of the custard powder (Jay 2000; Adams and Moss 2005).

**Table 5:** Analysis of variance of the effect of storage on the microbiological count of Cassava-based Custard Powder

Parameters	Total bacterial count (×10^4^ cfu/g)	Total yeast count (×10^4^ cfu/g)	Total mold count (×10^4^ cfu/g)
Storage period (Weeks)			
0	15.05 (6.73)^i^	0.41 (0.69)^i^	0.30 (0.42)^i^
3	17.82 (7.06)^h^	1.16 (0.97)^h^	1.02 (0.96)^h^
6	20.34 (7.14)^g^	1.95 (1.72)^g^	1.72 (1.14)^g^
9	24.82 (7.38)^f^	2.91 (2.86)^f^	2.39 (1.30)^f^
12	28.91 (7.43)^e^	4.09 (4.19)^e^	4.07 (2.22)^e^
15	32.66 (7.70)^d^	5.39 (4.24)^d^	5.59 (3.35)^d^
18	35.50 (8.97)^c^	6.89 (4.50)^c^	8.73 (5.54)^c^
21	40.93 (9.78)^b^	8.57 (5.28)^b^	10.73 (5.44)^b^
24	46.09 (9.91)^a^	12.73 (4.95)^a^	15.80 (6.29)^a^
Blend ratios			
94% YfCRS: 0.34% WEP	38.72 (9.01)^a^	2.44 (2.17)^gh^	4.92 (5.33)^f^
90% YfCRS: 2% WEP	29.64 (15.20)^c^	2.61 (3.61)^g^	5.31 (4.89)^e^
98% YfCRS: 2% WEP	29.94 (9.88)^c^	3.78 (3.56)^f^	4.56 (3.90)^g^
88.34% YfCRS: 6% WEP	28.61 (8.16)^d^	4.56 (4.04)^d^	2.50 (2.71)^h^
94% YfCRS: 6% WEP	39.06 (15.30)^a^	2.28 (2.63)^h^	4.83 (5.49)^f^
99.66% YfCRS: 6% WEP	34.22 (7.97)^b^	3.83 (3.32)^f^	5.61 (6.11)^d^
90% YfCRS: 10% WEP	24.56 (12.28)^e^	4.22 (4.59)^e^	5.89 (4.35)^c^
98% YfCRS: 10% WEP	23.00 (6.29)^f^	7.33 (6.58)^c^	8.97 (7.44)^b^
94% YfCRS: 11.66% WEP	24.61 (11.54)^e^	8.94 (6.54)^b^	5.83 (4.92)^cd^
TMS01/1368YfCRS	33.89 (11.89)^b^	2.36 (1.79)^gh^	2.17 (2.11)^i^
WEP	14.11 (8.08)^g^	11.53 (6.06)^a^	10.94 (10.57)^a^
Mean	29.12	4.90	5.59
CV (%)	3.19	10.66	7.74
*P* ratios	***	***	***
*P* storage	***	***	***
*P* storage × ratios	***	***	***

*** *P* ≤ 0.001, ()-standard deviation, cfu/g-Colony form unit per gram.

Means with different superscript on the same column are significantly different at *P* ≤ 0.05.

However, the total bacterial count of the custard powder after 24 weeks of storage was within the recommended limit prescribed by the International Microbiological Standards Recommended limit of bacteria contaminants for food of less than 10^6^ cfu/g as well as that of the center for food safety (10^5^ to <10^6^ cfu/g) (Shobha et al. 2011). Conversely, the mold and yeast count of the CbCP were higher than 10^3^ cfu/g prescribed by the International Microbiological Standards Recommended limit (Shobha et al. 2011). Though, the result of this research was within the range of values (10^5^–10^6 ^cfu/g) reported by Aran and Eke (1987) on the mold count in cereal samples in Turkey.

### Effect of storage on the sensory attributes of cassava starch-based custard powder

The sensory attributes of the stored CbCP prepared into gruel was significantly (*P* ≤ 0.001) affected by the blend ratios and storage periods, while the interactions between the blend ratios and the storage period had no significant effect (*P* *>* 0.05) (Table6). This implied that all the sensory perception of the custard gruel was considerably influenced by the combination of YfCRS and WEP in the custard formulation as well as the changes in temperature and relative humidity of the storage condition. Out of all the sensory attributes of the CbCP gruel evaluated, only the taste and color were disliked at the end of the storage, while the flavor, appearance, and mouth-feel were still within the likeness range (Table6). The dislike of the taste might be due to absorption of moisture by the custard powder during storage. This moisture absorption might have stimulated the activity of lipase and thus, splits up fat into free fatty acids and glycerol, which may cause rancidity (Akhtar et al. 2005). However, the overall sensory acceptability of the CbCP gruel was based on its flavor, appearance and mouth-feel, with custard powder produced from 94% YfCRS: 0.34% WEP and 90% YfCRS: 2% WEP blends having the highest acceptability at the end of storage (Table6).

**Table 6:** Analysis of variance of the effect of storage on the sensory properties of Cassava-based Custard Powder prepared into gruel

Parameters	Taste	Color	Flavor	Appearance	Mouth-feel	Overall acceptability
Storage						
0 Week	6.86 (1.29)^a^	6.58 (1.29)^a^	6.72 (1.62)^a^	6.70 (1.23)^a^	6.79 (1.30)^a^	6.68 (1.18)^a^
6 Weeks	6.86 (1.29)^a^	5.60 (1.24)^b^	6.66 (1.70)^a^	6.66 (1.33)^a^	6.77 (1.30)^a^	6.63 (1.28)^a^
12 Weeks	5.98 (1.14)^b^	4.60 (1.24)^c^	6.67 (1.68)^a^	6.66 (1.33)^a^	6.80 (1.27)^a^	6.67 (1.18)^a^
18 Weeks	5.30 (0.97)^c^	4.86 (1.29)^c^	6.63 (1.76)^a^	5.70 (1.23)^b^	6.80 (1.27)^a^	6.67 (1.18)^a^
24 Weeks	5.05 (0.84)^d^	3.97 (1.17)^d^	5.81 (1.43)^b^	5.67 (1.30)^b^	5.87 (1.14)^b^	5.68 (1.19)^b^
Blend ratios						
94% YfCRS: 0.34% WEP	6.74 (1.56)^a^	6.14 (1.60)^a^	6.90 (1.71)^ab^	7.70 (0.86)^a^	6.80 (1.34)^a-c^	6.90 (1.51)^a^
90% YfCRS: 2% WEP	6.06 (1.46)^b-d^	5.18 (1.52)^bc^	6.82 (1.81)^ab^	6.40 (1.20)^b^	6.90 (1.93)^ab^	7.02 (1.49)^a^
98% YfCRS: 2% WEP	5.84 (1.17)^b-d^	5.08 (1.37)^b-d^	5.68 (1.85)^d^	6.40 (1.63)^b^	6.82 (1.29)^a-c^	6.80 (0.88)^ab^
88.34% YfCRS: 6% WEP	5.62 (1.68)^e^	4.88 (1.91)^c-e^	6.90 (1.58)^ab^	5.92 (1.47)^cd^	6.44 (1.62)^bc^	6.18 (1.47)^c^
94% YfCRS: 6% WEP	6.18 (1.56)^b^	5.02 (1.44)^b-d^	6.62 (1.83)^ab^	6.46 (1.61)^b^	7.16 (1.02)^a^	6.36 (1.66)^bc^
99.66% YfCRS: 6% WEP	5.56 (0.99)^e^	4.42 (1.13)^e^	6.44 (1.62)^a-c^	5.50 (0.86)^d^	6.40 (0.78)^c^	6.10 (0.99)^c^
90% YfCRS: 10% WEP	5.64 (1.19)^de^	4.62 (1.16)^de^	6.26 (1.43)^b-d^	5.60 (0.93)^d^	5.62 (0.92)^d^	6.30 (0.65)^c^
98% YfCRS: 10% WEP	5.70 (0.97)^c-e^	5.32 (1.41)^bc^	6.92 (1.54)^a^	6.20 (1.05)^bc^	6.50 (0.89)^bc^	6.12 (1.14)^c^
94% YfCRS: 11.66% WEP	6.08 (1.56)^bc^	5.42 (1.57)^b^	5.94 (1.22)^cd^	6.30 (1.22)^bc^	6.80 (0.99)^a-c^	6.40 (0.78)^bc^
Mean	5.94	5.12	6.5	6.28	6.60	6.46
CV (%)	18.7	23.29	25.56	18.84	18.84	18.69
*P* blend ratios	***	***	***	***	***	***
*P* storage	***	***	***	***	***	***
*P* storage × blend ratios	NS	NS	NS	NS	NS	NS

*** *P* ≤ 0.001; (), Standard deviation; NS, Not significant.

Means with different superscript on the same column are significantly different at *P* ≤ 0.05.

## Conclusion

The protein, fat, and the total *β*-carotene contents of the CbCP reduced significantly (*P* ≤ 0.001) after storage while moisture content and microbiological load increased with storage time. The protein and fat content of the stored CbCP significantly correlated with the bacterial (*r* = −0.61, *P* ≤ 0.01) and yeast (*r* = 0.74, *P* ≤ 0.01) counts. Additionally, it was color and taste that deteriorated out of all the sensory attributes at the end of storage. However, custard gruel from 94% YfCRS: 0.34% WEP and 90% YfCRS: 2% WEP was generally accepted at the end of the 24 weeks of storage.
